# The pattern of gestational weight gains among Chinese women: a repeated measure analysis

**DOI:** 10.1038/s41598-018-34227-8

**Published:** 2018-10-26

**Authors:** Jing Tan, Yan Ren, Yana Qi, Peng Chen, Li Tang, Guolin He, Sheyu Li, Xin Sun, Xinghui Liu

**Affiliations:** 1 Chinese Evidence-based Medicine Center and CREAT Group, West China Hospital, Sichuan University, Chengdu, China; 2 West China Women and Children’s Hospital, Sichuan University, Chengdu, China; 3 Department of Endocrinology and Metabolism, West China Hospital, Sichuan University, Chengdu, China

## Abstract

This study aimed to investigate the pattern of gestational weight gain (GWG) among Chinese women. We included pregnant women, who delivered at a referral medical center in China and had no pre-specified adverse pregnancy outcomes. We collected weight data across all pregnancy visits, and used the two-level spline linear model to fit for the pattern of GWG according to pre-pregnancy BMI categories. In total, 4,567 pregnant women with 47,699 repeated measures were eligible. For those who were underweight, normal and overweight before pregnancy, the interquartile ranges (25^th^ to 75^th^ quartiles) of GWG between 14 and 36 gestational weeks were 12.9–17.7 kg, 12.6–17.3 kg and 11.7–16.8 kg, and the corresponding rates of GWG were 2.62 kg/month, 2.56 kg/month and 2.37 kg/month. For underweight and normal women, the rates of GWG were similar before 14 weeks (0.57 and 0.58 kg/month) and after 36 weeks (1.69 and 1.70 kg/month); however, the rates of GWG were higher for overweight women (0.66 kg/month before 14 weeks and 1.89 kg/month after 36 weeks). In summary, the pattern of GWG among Chinese pregnant women is generally consistent with the IOM recommendation, particularly for those who are normal or underweight. Modifications are, however, warranted for overweight pregnant women.

## Introduction

Gestational weight gain (GWG) is closely associated with many adverse neonatal and maternal outcomes. For example, excessive GWG often leads to higher risk of gestational diabetes, cesarean delivery, large-for-gestational-age babies, postpartum weight retention, and childhood obesity^1–4^. Insufficient weight gain may increase the risk of preterm birth, small-for-gestational-age babies, and fetal death^5–7^. Overweight and obesity at childbearing age represent a public health concern^8,9^. Monitoring maternal weight change has become a regular practice in many countries^10^.

The U.S. Institute of Medicine (IOM) released the guideline for managing weight gain and nutrition during pregnancy in 1990^11^ and an updated version in 2009^12^, both of which offered recommendations about gestational weight gains for women with different body mass index (BMI) before pregnancy. Currently, the 2009 guideline has been widely used in western countries^10,13^, and is also used for managing pregnancy weight among the Chinese population^14,15^.

However, the IOM guideline was developed primarily with Caucasian and black population^12^. There are important differences in anthropometry, culture and lifestyles between the U.S. and the Chinese population. For instances, the American women are generally higher than the Chinese women^16^; the intake of energy and total fat is higher as well^17^. Influenced by traditional Chinese culture, pregnancy is a much attended topic, and a number of changes may take place on behaviors and dietaries during pregnancy^18^. There are also differences in the medical care on pregnancy between the U.S. and the Chinese populations. For instance, pregnant women, especially those with excessive weight gain, are usually required to control weight starting 36 gestational weeks in China (but not in the U.S. population).

Because of these differences, the pattern of weight gain during pregnancy may differ between the U.S. and the Chinese population. Earlier evidence has suggested that the IOM guideline, may not be applicable to the Chinese population. One study, including singleton pregnant women in Shanghai, found that the IOM recommendation was probably not optimal for the Chinese population^19^. Another study from Taiwan showed that, using the IOM recommendation, women with normal weight and underweight had increased risk of low birth weight^20^.

Despite these findings, the pattern of GWG in the Chinese population has not yet been well defined, and the applicability of IOM recommendation to the Chinese population has not been confirmed. The lack of population-specific evidence may have made the management of maternal weight gains less effective. Given the different anthropometric and lifestyles compared with those among women included in IOM recommendation, it would thus be highly desirable to investigate the pattern of GWG in the Chinese pregnant women. The resulting evidence may inform the decision as to whether population-specific recommendations for GWG in China are needed.

## Materials and Methods

### Study setting

Our study was conducted at the West China Women and Children’s Hospital of Sichuan University in Chengdu, China. The hospital is a referral medical center in the southwest part of China and has more than 7000 deliveries each year. Usually, pregnant women were registered between 10 and 15 gestational weeks, and an electronic file was set up at the first antenatal visit. Thereafter, they conducted antenatal visits every two to four weeks until delivery.

At each visit, the information regarding medical history, co-morbidities, physical examination, imagining examination, and medical advice and prescription were recorded by nurses and doctors. The information about demographics and pre-pregnancy condition was recorded at the first visit. The admission and discharge information, such as maternal outcomes and neonatal outcomes, were also documented. All the information was stored in electronic medical records.

This study is a retrospective observational study. We used medical records of patients, without involving patients during conduct of the study and collection of data. The study was exempt from patient informed consent based on the ethic approval of Medical Ethics Committee of West China Second Hospital, Sichuan University (Approval number: 2016-028). The study data may be available upon request.

### Eligibility Criteria

We included the pregnant women who delivered between January 1, 2013 and December 31, 2014; had gestational age between 37 and 41 weeks; were registered at the first prenatal visit before 15 gestational weeks; had at least five follow-up visits until delivery; and were singleton pregnancy. We excluded those who developed pre-specified adverse outcomes during pregnancy, or those who had history of bariatric surgery.

In order to pre-specify adverse outcomes for pregnant women and newborns, we convened a group of experts, consisting of five obstetricians (two professors and three attending physicians in Obstetrics), one nutritionist (professor of maternal and child nutrition) and two epidemiologists (a professor and a lecturer). The maternal and neonatal adverse outcomes included preeclampsia, severe preeclampsia, eclampsia, HELLP syndrome, intrahepatic cholestasis at pregnancy, placenta praevia, placental abruption, gestational diabetes, postpartum hemorrhage, admission more than 7 days, ICU admission, postpartum infection, premature rupture of fetal membranes, birth weight more than 4000 g, birth weight less than 2500 g, large for gestational age, small for gestational age, newborn death, stillbirth, admission neonatal ICU, and newborn defects.

### Data Collection

We used pre-defined and pilot-tested Case Report Form (CRF) to collect data. Prior to the start of study, all the research staffs received training to understand the study protocol and standard operation procedures. They manually collected the information about weight at each prenatal visit from medical charts, and other information from electronic medical records. All the data were double-entered to check for consistency. The information missed in electronic medical records was recorded as missing values; any errors judged by attending physicians in Obstetrics were also deemed as missing values, if confirmed.

The study variables included demographics, maternal anthropometry, history of diseases, history of gestation and birth, gestational co-morbidities, maternal outcomes and neonatal outcomes. Demographic data were maternal age, location, education level, gravidity, parity, use of *in vitro* fertilization (IVF) and gestational age. Gestational age was clinically measured since the last menstrual period and verified by the first trimester ultrasound of fetal size when needed. The maternal anthropometry included height, pre-pregnancy weight, and weight at each prenatal visit and weight just before delivery. Height was measured at the first prenatal visit with accuracy of 1 centimeter. Pre-pregnancy weight was self-reported by pregnant women and follow-up weight data were measured at each prenatal visit by standard weight scale with accuracy of 0.1 kilogram; weight before delivery was available from the admission examination records. The time (gestational week) and the number of weight measurements varied in individual records due to variations in the prenatal visits. Laboratory tests included 75 g oral glucose tolerance test (OGTT), complete blood count, liver function, renal function, and ferritin. Gestational co-morbidities consisted of hematological diseases, cardiovascular diseases, digestive diseases, endocrine diseases, respiratory diseases, immune diseases, reproductive diseases, and mental and neurological disease.

### Data analysis

We used descriptive statistics to summarize demographic and gestational characteristics, including maternal age, height, education, location, gravidity, parity, use of IVF, gestational age, birth weight, birth length, and the number of prenatal visits. The pre-pregnancy BMI (kg/m^2^) was calculated by pre-pregnancy weight (kilogram) divided by square of height (meter). We defined pre-pregnancy BMI according to WHO standard for Asian population (underweight, normal weight, overweight and obesity: <18.5, 18.5–23.0, 23.0–27.5, and ≥27.5 kg/m^2^)^21^. The GWG was calculated by subtracting pre-pregnancy weight from weight measured at each prenatal visit.

The repeated measurements of weight gain formed a multilevel structure, where level 1 was the weight data measured repeatedly at each prenatal visit within an individual, and level 2 was an individual pregnant woman. We hypothesized that the pattern of weight gain was piecewise linear, because of the following: (1) weight gain usually increased slowly during the first trimester (before the 14 gestational weeks) and the current IOM guideline (2009) recommends different rates of gestational weight gains between the first versus the second/third trimesters; (2) pregnant women are usually required to control weight starting 36 gestational weeks in China, by which the speed of weight gain may change.

We thus constructed a two-level linear spline model, as below, to describe the pattern of gestational weight gain. The multi-level spline model has been well discussed and examined earlier^22^, and also has been successfully used in previous studies describing pattern of gestational weight gains^23–25^.

$${y}_{ij}={\beta }_{0}+\sum _{l=1}^{m}({\beta }_{l}+{u}_{lj}){T}_{lij}+{\beta }_{m+n}{X}_{nj}+{\beta }_{m+n+{l}^{\ast }n}\sum _{l=1}^{m}{T}_{lij}\times {X}_{nj}+{u}_{0j}+{\varepsilon }_{0ij}$$

$$[\begin{array}{c}{u}_{0j}\\ {u}_{lj}\end{array}]:\,N(0,{{\rm{\Omega }}}_{u}),{{\rm{\Omega }}}_{u}=[\begin{array}{c}{\sigma }_{u0}^{2}\\ {\sigma }_{u0l}\,{\sigma }_{ul}^{2}\end{array}]$$

$${\varepsilon }_{0ij}:\,N(0,{{\rm{\Omega }}}_{\varepsilon }),{{\rm{\Omega }}}_{\varepsilon }={\sigma }_{\varepsilon 0}^{2}$$

where i indexed repeated measurement in at level 1, j indexed the pregnant women at level 2, *y*_*ij*_ was the GWG for the i gestational age in the j pregnant woman, *T*_*lij*_ was the gestational age spline, and *X*_*nj*_ was the pre-pregnancy BMI categories. The parameter β_0_ estimated the mean GWG of the reference BMI group, the parameter β_*m* + *n*_ estimated the difference GWG between the reference BMI group and other BMI group, the parameter β_*l*_ estimated the mean change rate of the reference BMI group at the gestational age spline, the parameter β_*m*+n+l*n_ estimated the different change rate between the reference BMI group and other BMI group at the gestational age spline. M means the number of parts in spline, *u*_0*j*_ for random effect in level 2, *u*_*lj*_ for random effect of mean change GWG in level 2 and *ε*_0*ij*_ for residual error. By estimating and testing variance terms ${\sigma }_{u0}^{2}$ and ${\sigma }_{ul}^{2}$ of the random effects *μ*_0*j*_ and *μ*_*lj*_ respectively, we can find out the distribution of the GWG and gestational age random effects at individual level.

To examine the hypothesized knots (T_lij) in the spline model, we first plotted the line chart of mean GWG by gestational age according to pre-pregnancy BMI. We then constructed the two-level spline model that minimized the difference between predicted and original values of GWG. To examine the statistical performance, we used the Akaike’s Information Criterion (AIC) for the model choice.

We plotted the estimated trajectories and actual observations by gestational weeks, and presented smoothed percentiles (5^th^, 25^th^, 50^th^, 75^th^, and 95^th^ percentile) for GWG by gestational weeks, both according to pre-pregnancy BMI categories. These percentiles were calculated using a statistical method previously discussed^26^. We compared the estimated and observed values at the 5^th^, 25^th^, 50^th^, 75^th^ and 95^th^ percentiles for each of the weight groups.

In order to compare GWG recommended by the IOM guideline, we reported interquartile range (25^th^ to 75^th^ percentile) and 90% percentile range (5^th^ to 95^th^ percentile) of GWG according to pre-pregnancy BMI. Derived from the parameters of regression model, we calculated rates of GWG at each part of spline.

We conducted a sensitivity analysis by adjusting for potential confounders in regression model to examine the robustness of our results. The confounders included baseline characteristics with statistical significance (*p* < 0.05). We also undertook another sensitivity analysis by using pre-pregnancy BMI cut-offs according to IOM guideline to develop the ranges and rates of GWG. We used package MLwiN 2.30 for the modeling analysis and SAS 9.4 for descriptive analysis.

## Results

**Figure 1:**
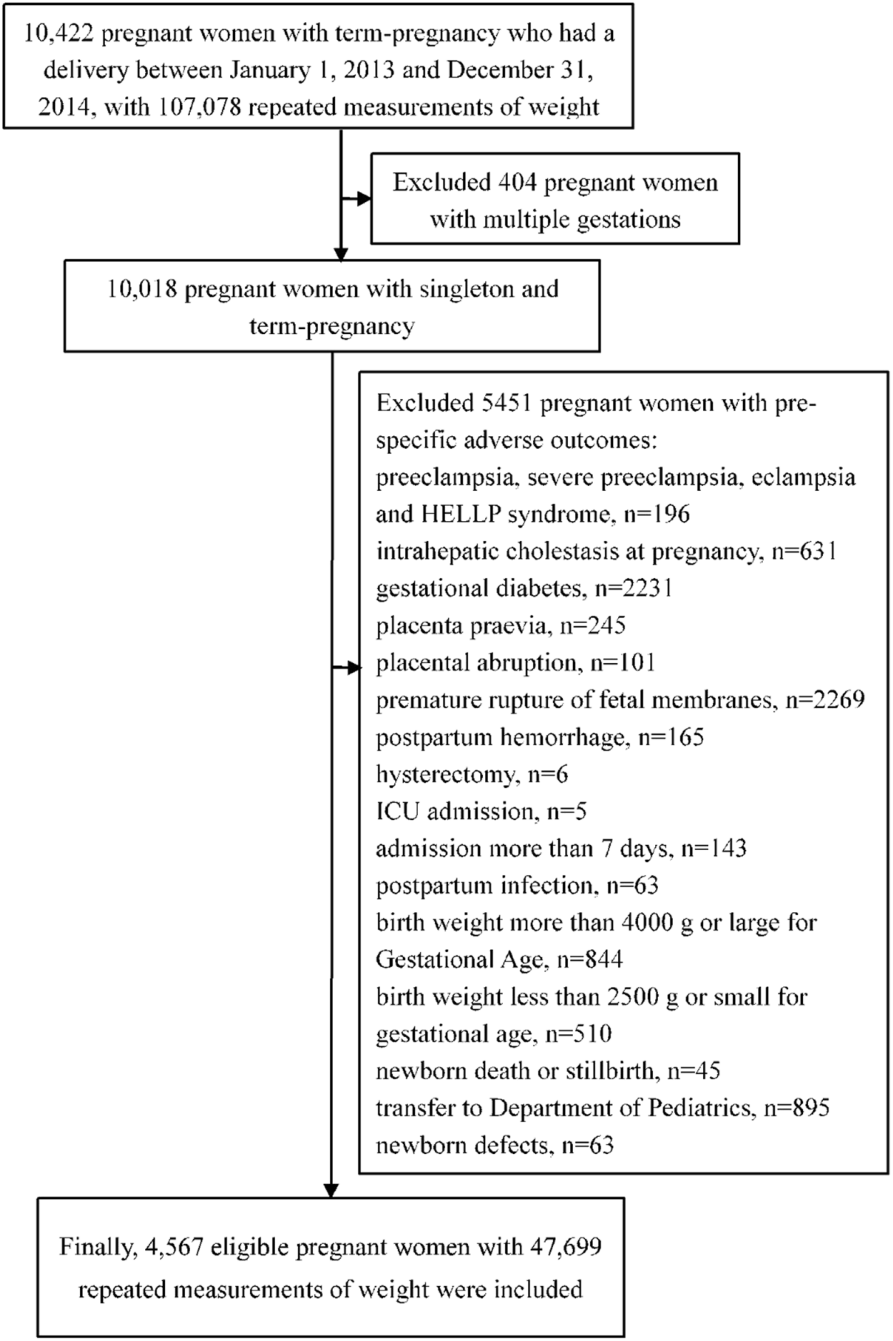
The flow chart of included population.

We initially collected data from 10,422 pregnant women who delivered between January 1, 2013 and December 31, 2014. Among those, 5451 individuals developed one of pre-specified adverse outcomes. Gestational diabetes and premature rupture of fetal membranes were the most frequent adverse outcomes. As a result, 4,567 eligible pregnant women, consisting of 47,699 repeated measurements of weight data, were eligible (Fig. 1). Because only 53 pregnant women were obese (BMI ≥ 27.5 kg/m^2^), we excluded this subset of population from analysis. Finally, 4,514 pregnant women were included for analysis. No missing data was present in our analysis.

### Characteristics of pregnant women

Among those 4,514 individuals, 3017 (66.8%) had normal weight before pregnancy, 763 (16.9%) were underweight, and the rest were overweight (16.3%) according to the WHO BMI standard for Asian population. Each woman had a mean of 10.45 (SD 1.94) measurements of weight; about 90% received higher education (≥12 education years), and 94.2% of pregnant women resided in urban areas. The mean maternal age was 30.13 (SD 3.95) years, and the mean gestational age was 39.19 (SD 0.95) week; 3693 (81.81%) were primipara; the median of gravidity was 2 (interquartile range 1–3); 118 (2.6%) pregnant women used IVF. The mean pre-pregnancy BMI was 20.69 (SD 2.29) kg/m^2^. By the univariable analysis, we found the maternal age, education level, gravidity, parity, use of IVF, birth weight, birth height and gestational age at delivery were different among pre-pregnancy BMI categories (*p* < 0.05) (Table 1).

**Table 1:** Demographic and obstetric characteristics of pregnant women according to pre-pregnancy BMI categories.

Characteristics	All (n = 4514)^†^	Underweight (n = 763)	Normal weight (n = 3017)	Overweight (n = 734)	*P* value
No. individual (%)	4514 (100.00)	763 (16.90)	3017 (66.84)	734 (16.26)	—
No. repeated measurements (times) (%)	47169 (100.00)	7924 (16.80)	31567 (66.92)	7678 (16.28)	—
Mean repeated measurements (times) (SD)	10.45 (1.94)	10.39 (1.82)	10.45 (1.95)	10.52 (2.09)	0.612
Maternal age (years) (SD)	30.13 (3.95)	28.84 (3.57)	30.17 (3.90)	31.32 (4.11)	<0.0001
Maternal height (meter) (SD)	1.61 (0.05)	1.61 (0.05)	1.61 (0.05)	1.60 (0.05)	0.045
Pre-pregnancy BMI (kg/m^2^) (SD)	20.69 (2.29)	17.54 (0.78)	20.58 (1.20)	24.42 (1.11)	<0.0001
**Education (%)**					
Education years >12 years	4057 (89.88)	692 (90.69)	2743 (90.92)	622 (84.74)	<0.0001
Education years ≤ 12 years	457 (10.12)	71 (9.31)	274 (9.08)	112 (15.26)	
**Location (%)**					
City	4253 (94.22)	722 (94.63)	2839 (94.10)	692 (94.28)	0.854
Rural	261 (5.78)	41 (5.37)	178 (5.90)	42 (5.72)	
Gravidity (times) (median, interquartile)	2 (1–3)	1 (1–2)	2 (1–3)	2 (1–3)	0.0001
**Parity**					
Nulliparity	3693 (81.81)	662 (86.76)	2472 (81.94)	559 (76.16)	<0.0001
Multiparity	821 (18.19)	101 (13.24)	545 (18.06)	175 (23.84)	
**Use of IVF**					
Yes	118 (2.61)	10 (1.31)	70 (2.32)	38 (5.18)	<0.0001
No	4396 (97.39)	753 (98.69)	2947 (97.68)	696 (94.82)	
Birth weight (kg) (SD)	3.31 (0.32)	3.23 (0.32)	3.31 (0.32)	3.40 (0.31)	<0.0001
Birth height (cm) (SD)	49.90 (1.51)	49.68 (1.44)	49.91 (1.54)	50.07 (1.42)	<0.0001
Gestational age at delivery (SD)	39.19 (0.95)	39.10 (0.95)	39.21 (0.95)	39.17 (0.95)	0.011

Notes: ^†^Excluding 53 pregnant women with pre-pregnancy obesity (pre-pregnancy BMI ≥ 27.5 kg/m^2^).

Abbreviations: BMI, body mass index; SD, standard deviation; IVF, *in vitro* fertilization.

### Gestational weight gains and the two-level spline model

**Figure 2:**
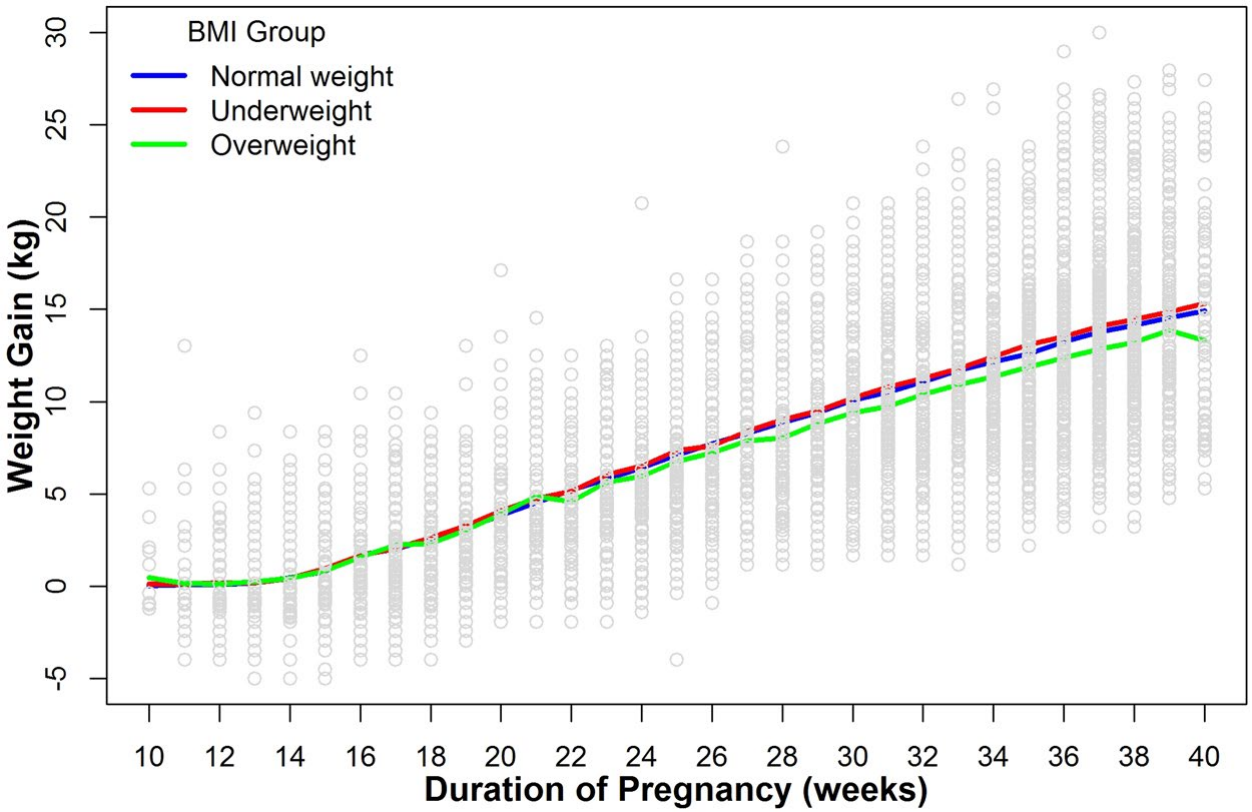
Line chart of mean GWG by gestational age according to the pre-pregnancy BMI categories. Blue line for pregnant women with pre-pregnancy normal weight, red line for pregnant women with pre-pregnancy underweight, and green line for pregnant women with pre-pregnancy overweight.

Figure 2 showed the observed GWG by gestational weeks according to pre-pregnancy BMI. The pattern of mean weight gains suggested that the speed of weight gain appeared to have changed at 14 gestational weeks, and there was a slight change at 36 weeks (given the regular medical interventions on weight gain at that time in China). The use of AIC statistics suggested that the statistical model with the two knots (i.e. 14 and 36 weeks) performed better (AIC: 147775 with the knot at 14 and 36 weeks versus 150283 with knot at 14 weeks versus 154927 without knots).

Figure S1 presented the estimated trajectories and observed data by gestational weeks according to pre-pregnancy BMI. For the underweight and normal groups, the estimated and observed values at the 5^th^, 50^th^ and 95^th^ percentiles were very close after 14 weeks, but not between 10 and 14 weeks. For the overweight group, there were deviations at some time points between the estimated and observed values.

### Estimated pattern of gestational weight gain

**Figure 3:**
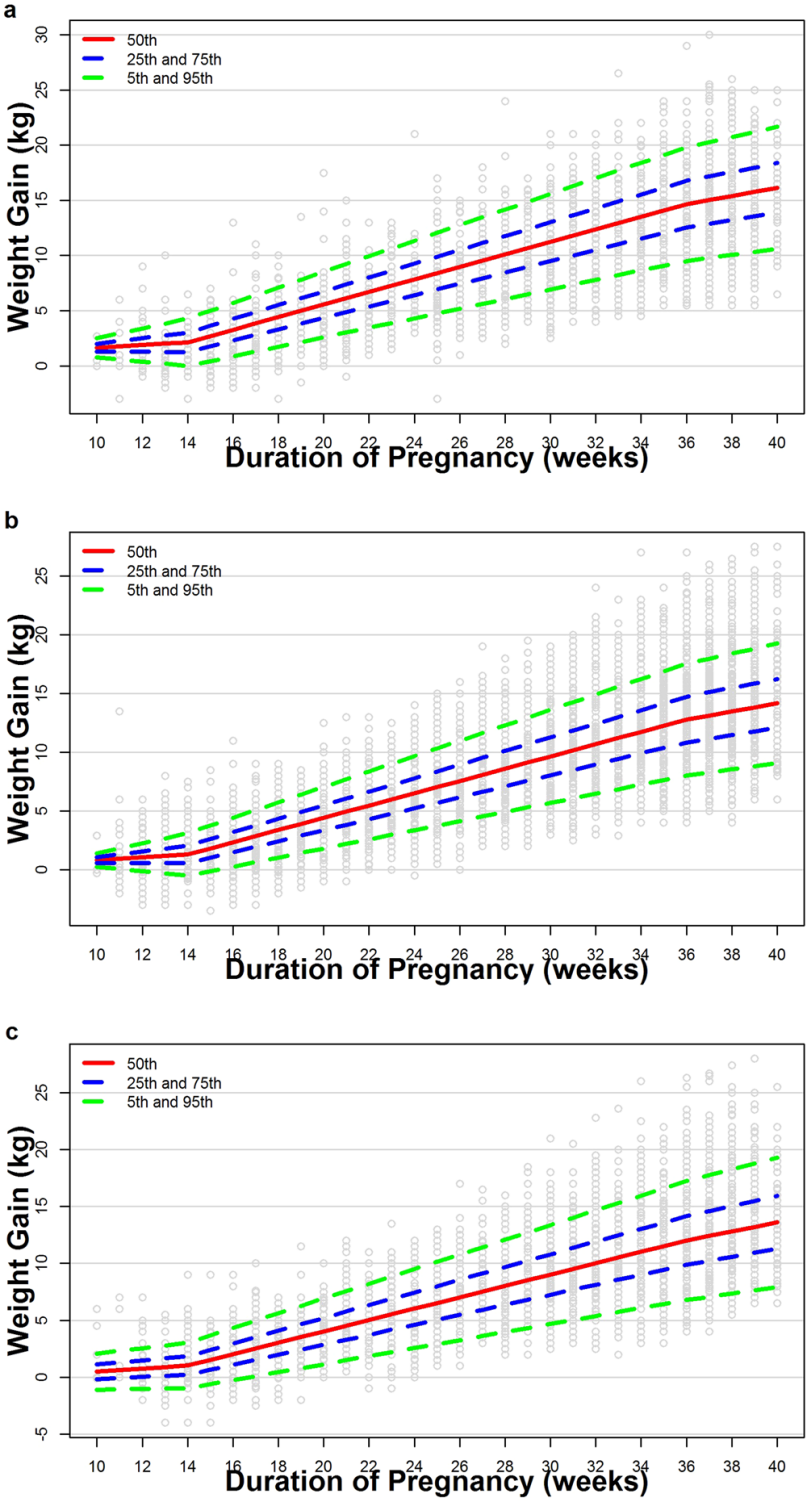
(**a**–**c**) Estimated trajectories at 5th, 25th, 50th, 75th, and 95th centiles of weight over gestational weeks among underweight (**a**), normal weight (**b**), and overweight (**c**) women. Small grey circles are actual observations.

Tables S1–S3 and Fig. 3 presented the estimated trajectories for GWG at the 5^th^, 25^th^, 50^th^, 75^th^, and 95^th^ percentiles by gestational weeks according to pre-pregnancy BMI. The interquartile ranges of weight gain among the underweight, normal and overweight women were 12.9 to 17.9 kg, 12.6 to 17.3 kg, and 11.7 to 16.8 kg; their corresponding 90% ranges (i.e. 5^th^ to 95^th^ percentile) were respectively 9.4 to 21.1 kg, 9.2 to 20.7 kg and 8.0 to 20.3 kg (Table 2).

**Table 2:** The ranges and rates of GWG stratified by pre-pregnancy BMI categories.

Pre-pregnancy BMI Category^a^	IOM recommendation of GWG (kg)^b^	P_25_–P_75_ GWG (kg)	P_5_–P_95_ GWG (kg)	Rates of GWG^c^ at 10–14 weeks (kg/month)	Rates of GWG^c^ at the 14–36 weeks (kg/month)	Rates of GWG^c^ after 36 weeks (kg/month)
Underweight	12.5–18	12.9–17.7	9.4–21.1	0.57	2.62	1.69
Normal weight	11.5–16	12.6–17.3	9.2–20.7	0.58	2.56	1.70
Overweight	7–11.5	11.7–16.8	8.0–20.3	0.66	2.37	1.89

Note: ^a^WHO Asian BMI standard is used for developing the ranges and rates of GWG, the pre-pregnancy BMI cut-offs for underweight, normal weight and overweight are <18.50 kg/m^2^, 18.50–22.99 kg/m^2^, 23.00–27.49 kg/m^2^ respectively.

^b^In IOM recommendation, the pre-pregnancy BMI cut-offs for underweight, normal weight and overweight are <18.5 kg/m^2^, 18.5–24.9 kg/m^2^, 25.0–29.9 kg/m^2^ respectively.

^c^The rates of GWG at the 10–14 weeks (kg/week) are the coefficients of T1 of regression equation in Table 3 (kg/month = kg/week*4.34524), the coefficients of T2 for the rates of GWG at the 14–36 weeks (kg/week), and the coefficients of T3 for the rates of GWG after the 36 weeks (kg/week).

Abbreviations: GWG, gestational weight gain; BMI, body mass index; IOM, Institute of Medicine.

Table 3 presented the equations of weight gain models according to pre-pregnancy BMI. Derived from the coefficients of models, the rates of GWG at 10–14 weeks were modest across all pre-pregnancy BMI categories (0.57–0.66 kg/month). At 14–36 weeks, the rates of GWG increased across all categories (underweight 2.62 kg/month; normal 2.56 kg/month; overweight 2.37 kg/month). At the last month of gestation, the rates slowed down to 1.69–1.89 kg/month across three groups.

**Table 3:** Regression models according to pre-pregnancy BMI categories.

Estimate	Regression model
**Underweight**	
Mean	−0.211 + 0.132*point1 + 0.604*point2 + 0.388*point3
SD	0.359 + 0.206*point1 + 0.088*point2 + 0.061*point3
**Normal weight**	
Mean	−0.259 + 0.134*point1 + 0.590*point2 + 0.392*point3
SD	0.325 + 0.187*point1 + 0.093*point2 + 0.069*point3
**Overweight**	
Mean	−0.246 + 0.151*point1 + 0.546*point2 + 0.434*point3
SD	1.529–0.054*point1 + 0.105*point2–0.051*point3

Note: Using equations of mean and SD one can compute any desired percentiles using relation P^th^ percentile = mean + K*SD, where K is normal equivalent deviate (z score) corresponding to particular percentile, for example, K = 1.64 for 95th percentile and −1.64 for 5th percentile, and SDs in this equation are predicted estimates from the regression model.

For example, to calculate GWG at 24 weeks for normal weight pregnant woman:

p50 for GWG = (−0.259 + 0.134*5 + 0.590*10 + 0.392*0) + (0 × (0.325 + 0.187*5 + 0.093*10 + 0.069*0)).

p5 for GWG = (−0.259 + 0.134*5 + 0.590*10 + 0.392*0) + (−1.64 × (0.325 + 0.187*5 + 0.093*10 + 0.069*0)).

p95 for GWG = (−0.259 + 0.134*5 + 0.590*10 + 0.392*0) + (1.64 × (0.325 + 0.187*5 + 0.093*10 + 0.069*0)).

Abbreviations: BMI, body mass index; SD, standard deviation.

The sensitivity analysis that adjusted for potential confounding factors showed similar findings (Table S4). The sensitivity analysis using the IOM standard for pre-pregnancy BMI cut-offs suggested similar ranges of GWG in underweight and normal groups. However, the range of GWG in overweight group was lower than those developed by WHO Asian BMI standard (11.1–15.6 kg versus 11.7–16.8 kg) (Table S5).

## Discussions

Using repeated measure data, our study constructed the model for describing pattern of gestational weight gain among the Chinese women with different BMI categories before pregnancy. To the best of our knowledge, this is one of the largest longitudinal studies investigating GWG in Asia^27–29^.

The results suggested that the model generally performed well to capture the patterns of gestational weight gains among the Chinese pregnant women. We identified deviations of predicted values from observed data at the early gestational age (e.g. less than 14 weeks), mainly because the data before that time were fewer, and the variability in GWG was substantial at the first trimester due to early pregnancy symptoms. We also found that the model of GWG for overweight women did not perform optimally, primarily because medical interventions on weight gain were often implemented in this subpopulation.

Our model also suggested the speed of gestational weight gains may differ at different gestational weeks (i.e. <14 versus 14 to 36 versus >36 gestational weeks); the change was particularly obvious at 14 weeks. This was not well investigated in previous studies addressing GWG among the Chinese population^12,27,30,31^. The identification of 14 gestational weeks as the weight gain shifting point is consistent with the IOM recommendation. Although the change of speed was less obvious at 36 weeks, the inclusion of this point was legitimate for two reasons. First, the AIC statistics suggested an improved model performance by including the point. Second, probably more importantly, in the Chinese healthcare system, weight control is usually reinforced for pregnant women starting 36 weeks (but not in the U.S. population). The inclusion of the point would largely represent the practical variations between the two healthcare systems.

Derived from the model, our study obtained the ranges of weight gain during gestation. We found that, compared to the IOM recommendations^12^, the Chinese pregnant women had more weight gains if pre-pregnancy BMI was normal (25–75^th^ percentile: 12.6–17.3 kg in the Chinese versus 11.5–16 kg according to IOM); the weight gain was even more significant among those who were overweight before pregnant (25–75^th^ percentile: 11.7–16.8 kg versus 7–11.5 kg). However, the weight gain was similar to IOM recommendations if underweight before pregnant (25–75^th^ percentile: 12.9–17.7 kg versus 12.5–18 kg).

Our study also found that the resulting rates of GWG between 10 and 14 weeks were similar across pre-pregnancy BMI groups (0.57–0.66 kilograms/week); the findings were consistent with other studies^32,33^. However, the rates of GWG between 14 and 36 weeks were consistently higher in our study than the IOM recommendations regardless of the pre-pregnancy BMI (2.62 vs 2.22 kg/month in the underweight group, 2.56 vs. 1.83 kg/month normal, and 2.37 vs. 1.22 kg/month overweight). After 36 weeks, the rates were lower than the IOM recommendation among the underweight (1.69 vs. 2.22 kg/month) and normal (1.70 vs. 1.83 kg/month) women, but higher among the overweight women (1.89 vs. 1.22 kg/month)^12^.

In addition, one may note the apparent statistical differences in birth weight, birth height and gestational age at delivery according to pre-pregnancy BMI categories, likely due to the large sample size (Table 1). Nevertheless, they were very similar among different pre-pregnancy BMI categories, and not clinically important.

Our study has some strengths. Firstly, this is the largest study with repeated measurement data to address the pattern of GWG among Chinese population. Secondly, we used rigorous methods for conducting the study. Throughout the study, we involved obstetricians, epidemiologists and statisticians, aiming to maximize the quality of data and the reliability of findings. Finally, our study used the two-level spline model to fit the pattern of GWG according to pre-pregnancy BMI categories^22^. It addressed critical limitations in the previous studies, in which repeated measurements were not well utilized and projected trajectories about the weight gain were not available^31,34–36^.

Our study also has a few limitations. Firstly, our analyses were based on data from a single medical referral center, and most pregnant women were well educated. The generalizability of the results was thus probably limited. Secondly, the number of overweight women was small. This resulted in suboptimal modeling of the pattern of GWG. Third, our study did not include obese women. The results may not be applicable to this subpopulation. Finally, the misclassification bias and measurement bias may not be inevitable as result of data collection from medical records.

## Conclusion

In summary, our study, using the two-level spline model, depicted the pattern of GWG among Chinese population. The model performed generally well. The patterns of GWG were close to the IOM recommendation if the women were underweight before pregnant. However, if normal or overweight, the Chinese women often gain more weight than the IOM recommendation. The findings suggested that modifications of the IOM recommendations to suit for the Chinese pregnant women may be warranted, particularly for the overweight women.

## Electronic supplementary material

Supplementary Information 

## Data Availability

Data sharing is possible upon ethnical approval and mutual agreement on data authorship.
