# Supplementary material for: The pattern of gestational weight gains among Chinese women: a repeated measure analysis
Source: Sci Rep. 2018 Oct 26;8:15865. doi: 10.1038/s41598-018-34227-8 (PMC6203785; doi:10.1038/s41598-018-34227-8)
Supplement: Supplementary file 1 — Supplementary Information [file 41598_2018_34227_MOESM1_ESM.pdf]

The pattern of gestational weight gains among Chinese women: a repeated measure analysis

Jing Tan,[1] Yan Ren,[1] Yana Qi,[1] Peng Chen,[2] Li Tang,[1] Guolin He,[2] Sheyu Li,[3] Xin Sun,[1*] Xinghui Liu[2*]

Affiliations

[1] Chinese Evidence-based Medicine Center and CREAT Group, West China Hospital, Sichuan University, Chengdu, China

[2] West China Women and Children's Hospital, Sichuan University, Chengdu, China

[3] Department of Endocrinology and Metabolism, West China Hospital, Sichuan University, Chengdu, China

Corresponding author

Xinghui Liu and Xin Sun

Address: No.20, Section 3, Renmin South Road, Chengdu, China;

No.37 Guo Xue Xiang, Chengdu, China

Telephone: +86 13881883798; +86 18980606047

E-mail: xinghuiliu@163.com; sunx79@hotmail.com

Supplementary Information

Table S1-S3 Smoothed percentiles of GWG for pregnant women with pre-pregnancy underweight (BMI <18.50 kg/m^2), normal weight (BMI 18.50-22.99 kg/m^2) and overweight (BMI 23.00-27.49 kg/m^2), respectively.

Table S4 Sensitivity analysis of the ranges and rates of GWG stratified by pre-pregnancy BMI categories

Table S5 The ranges and rates of GWG using the IOM standard for pre-pregnancy

Figure S1(Figure S1a, S1b, S1c) The 5th (bottom dashed line), 50th (middle), and 95th (top) estimated trajectories for GWG among underweight (a), normal (b), overweight (c) women. Large blue circles show empirical values at each gestational week. Small grey circles represent actual observations

Table S1 Smoothed percentiles for GWG for women with pre-pregnancy underweight (BMI <18.50) according to gestational age

Gestational age (weeks)	No of measures	Percentiles for GWF (kg)				
		5th	25th	50th	75th	95th
10	23	-1.01	-0.46	-0.08	0.30	0.85
11	120	-1.21	-0.46	0.05	0.57	1.32
12	267	-1.42	-0.47	0.18	0.84	1.79
13	216	-1.62	-0.48	0.32	1.11	2.25
14	166	-1.83	-0.48	0.45	1.38	2.72
15	165	-1.37	0.06	1.05	2.04	3.47
16	219	-0.91	0.61	1.66	2.70	4.22
17	213	-0.45	1.15	2.26	3.37	4.97
18	195	0.01	1.70	2.86	4.03	5.72
19	153	0.47	2.24	3.47	4.69	6.47
20	161	0.93	2.79	4.07	5.36	7.22
21	182	1.38	3.33	4.67	6.02	7.96
22	168	1.84	3.88	5.28	6.68	8.71
23	162	2.30	4.42	5.88	7.34	9.46
24	174	2.76	4.96	6.49	8.01	10.21
25	273	3.22	5.51	7.09	8.67	10.96
26	162	3.68	6.05	7.69	9.33	11.71
27	178	4.14	6.60	8.30	10.00	12.46
28	245	4.60	7.14	8.90	10.66	13.21
29	223	5.06	7.69	9.51	11.32	13.95
30	324	5.52	8.23	10.11	11.99	14.70
31	321	5.97	8.78	10.71	12.65	15.45
32	316	6.43	9.32	11.32	13.31	16.20
33	270	6.89	9.87	11.92	13.98	16.95
34	419	7.35	10.41	12.52	14.64	17.70
35	405	7.81	10.96	13.13	15.30	18.45
36	573	8.27	11.50	13.73	15.96	19.20
37	658	8.56	11.85	14.12	16.39	19.68
38	589	8.85	12.20	14.51	16.82	20.17
39	302	9.13	12.54	14.90	17.25	20.66
40	82	9.42	12.89	15.29	17.68	21.15

Abbreviations: GWG, gestational weight gain; BMI, body mass index

Table S2 Smoothed percentiles for GWG for women with pre-pregnancy normal weight (BMI 18.50-22.99) according to gestational age

Gestational age (weeks)	No of measures	Percentiles for GWF (kg)				
		5th	25th	50th	75th	95th
10	113	-0.77	-0.39	-0.13	0.14	0.52
11	519	-0.98	-0.40	0.01	0.41	1.00
12	1278	-1.20	-0.41	0.14	0.69	1.48
13	1074	-1.41	-0.41	0.28	0.97	1.97
14	783	-1.63	-0.42	0.41	1.24	2.45
15	799	-1.19	0.10	1.00	1.89	3.19
16	926	-0.75	0.63	1.59	2.55	3.93
17	1027	-0.32	1.16	2.18	3.20	4.67
18	919	0.12	1.69	2.77	3.85	5.41
19	694	0.56	2.21	3.36	4.50	6.15
20	683	1.00	2.74	3.95	5.15	6.90
21	824	1.44	3.27	4.54	5.80	7.64
22	865	1.87	3.80	5.13	6.45	8.38
23	715	2.31	4.33	5.72	7.11	9.12
24	873	2.75	4.85	6.31	7.76	9.86
25	1164	3.19	5.38	6.89	8.41	10.60
26	751	3.63	5.91	7.48	9.06	11.34
27	779	4.07	6.44	8.07	9.71	12.08
28	1214	4.50	6.96	8.66	10.36	12.82
29	1015	4.94	7.49	9.25	11.01	13.56
30	1457	5.38	8.02	9.84	11.66	14.30
31	1443	5.82	8.55	10.43	12.32	15.04
32	1520	6.26	9.07	11.02	12.97	15.79
33	1212	6.70	9.60	11.61	13.62	16.53
34	1984	7.13	10.13	12.20	14.27	17.27
35	1952	7.57	10.66	12.79	14.92	18.01
36	2731	8.01	11.19	13.38	15.57	18.75
37	3006	8.31	11.54	13.77	16.00	19.24
38	2723	8.60	11.89	14.16	16.44	19.73
39	1534	8.90	12.25	14.56	16.87	20.21
40	474	9.20	12.60	14.95	17.30	20.70

Abbreviations: GWG, gestational weight gain; BMI, body mass index

Table S3 Smoothed percentiles for GWG for women with pre-pregnancy overweight (BMI 23.00-27.49) according to gestational age

Gestational age (weeks)	No of measures	Percentiles for GWF (kg)				
		5th	25th	50th	75th	95th
10	8	-1.82	-0.80	-0.10	0.61	1.63
11	37	-1.79	-0.70	0.05	0.81	1.89
12	89	-1.75	-0.59	0.21	1.01	2.16
13	76	-1.72	-0.49	0.36	1.20	2.43
14	67	-1.69	-0.39	0.51	1.40	2.70
15	59	-1.30	0.09	1.05	2.01	3.41
16	77	-0.92	0.57	1.60	2.63	4.11
17	67	-0.53	1.05	2.14	3.24	4.82
18	62	-0.14	1.53	2.69	3.85	5.53
19	50	0.24	2.01	3.24	4.46	6.23
20	54	0.63	2.49	3.78	5.07	6.94
21	51	1.01	2.97	4.33	5.68	7.64
22	65	1.40	3.46	4.88	6.29	8.35
23	52	1.79	3.94	5.42	6.91	9.06
24	80	2.17	4.42	5.97	7.52	9.76
25	76	2.56	4.90	6.51	8.13	10.47
26	47	2.94	5.38	7.06	8.74	11.17
27	56	3.33	5.86	7.61	9.35	11.88
28	105	3.72	6.34	8.15	9.96	12.59
29	77	4.10	6.82	8.70	10.57	13.29
31	111	4.49	7.30	9.24	11.19	14.00
32	107	4.87	7.78	9.79	11.80	14.70
33	95	5.26	8.26	10.34	12.41	15.41
34	151	5.65	8.74	10.88	13.02	16.12
35	134	6.03	9.22	11.43	13.63	16.82
36	208	6.42	9.70	11.97	14.24	17.53
37	225	6.80	10.19	12.52	14.85	18.23
38	205	7.11	10.57	12.95	15.34	18.80
39	97	7.42	10.95	13.39	15.83	19.36
40	32	7.72	11.33	13.82	16.32	19.92

Abbreviations: GWG, gestational weight gain; BMI, body mass index

Table S4 Sensitivity analysis of the ranges and rates of GWG stratified by pre-pregnancy BMI categories

**Pre-pregnancy BMI Category**	**P_{25}-P_{75} GWG (kg)**	**P_5-P_{95} GWG (kg)**	**Rates of GWG at 10-14 weeks (kg/month)**	**Rates of GWG at 14-36 weeks (kg/month)**	**Rates of GWG after 36 weeks (kg/month)**
Underweight	12.7-17.5	9.2-20.9	0.58	2.63	1.69
Normal weight	12.4-17.1	9.0-20.5	0.59	2.56	1.71
Overweight	11.4-16.6	7.7-20.3	0.65	2.37	1.89

Note: We adjusted for the influence of maternal age, education level, gravidity, parity and use of IVF.

Abbreviations: GWG, gestational weight gain; BMI, body mass index; IVF, in vitro fertilization

Table S5 The ranges and rates of GWG using the IOM standard for pre-pregnancy

**Pre-pregnancy BMI Category**	**P_{25}-P_{75} GWG (kg)**	**P_5-P_{95} GWG (kg)**	**Rates of GWG at 10-14 weeks (kg/month)**	**Rates of GWG at 14-36 weeks (kg/month)**	**Rates of GWG after 36 weeks (kg/month)**
Underweight	12.9-17.7	9.4-21.1	0.53	2.42	1.55
Normal weight	12.5-17.3	9.0-20.8	0.56	2.34	1.58
Overweight	11.1-15.6	7.9-18.8	0.35	2.01	1.86

Notes: IOM BMI standard is used for developing the ranges and rates of GWG, and the pre-pregnancy BMI cut-offs for underweight, normal weight and overweight are **<** 18.5 kg/m^2, 18.5-24.9 kg/m^2, 25.0-29.9 kg/m^2 respectively.

Abbreviations: GWG, gestational weight gain; BMI, body mass index

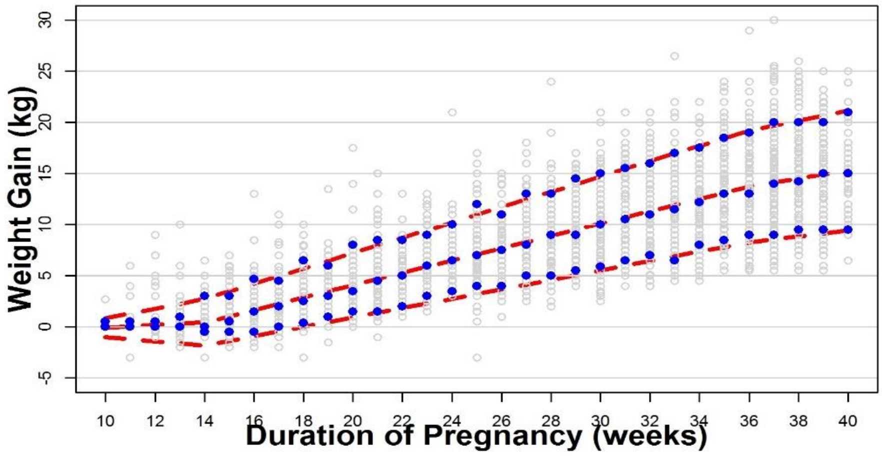

Figure S1a

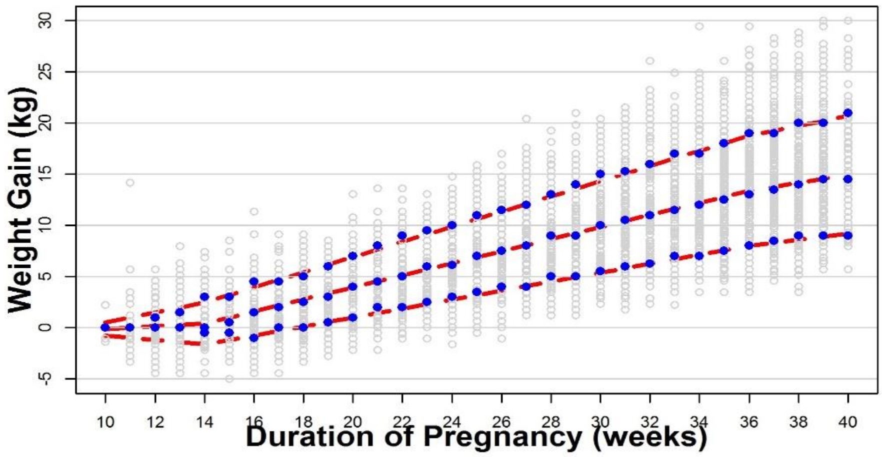

Figure S1b

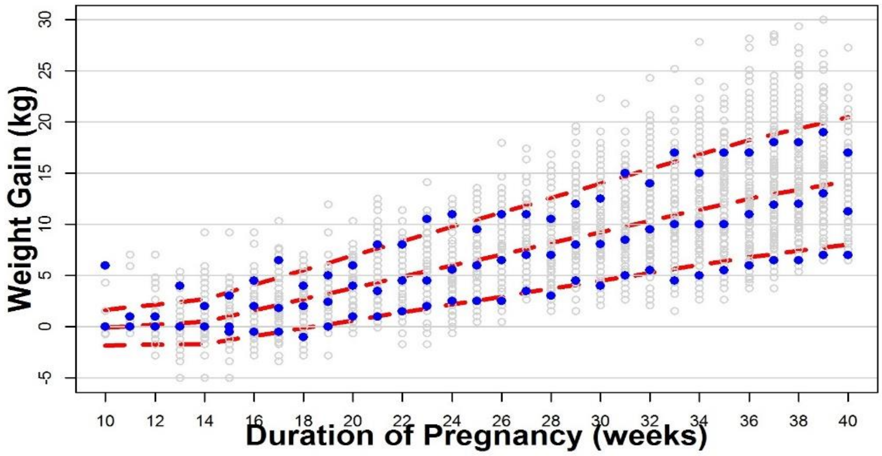

Figure S1c

Figure S1 The 5th (bottom dashed line), 50th (middle), and 95th (top) estimated trajectories for GWG among underweight (a), normal (b), overweight (c) women. Large blue circles show empirical values at each gestational week. Small grey circles represent actual observations
